# Associations between Metabolic Risk Factors and the Hypothalamic Volume in Childhood Leukemia Survivors Treated with Cranial Radiotherapy

**DOI:** 10.1371/journal.pone.0147575

**Published:** 2016-01-29

**Authors:** Cecilia Follin, Sanaz Gabery, Åsa Petersén, Pia C. Sundgren, Isabella Björkman-Burtcher, Jimmy Lätt, Peter Mannfolk, Eva Marie Erfurth

**Affiliations:** 1 Department of Endocrinology, Skåne University hospital and IKVL, Lund University, Lund, Sweden; 2 Translational Neuroendocrine Research Unit, Department of Experimental Medical Science, Lund University, Lund, Sweden; 3 Department of Diagnostic Radiology, Skåne University Hospital and IKVL/Lund University, Lund, Sweden; 4 Lund University Bioimaging Centre (LBIC), Lund University, Lund, Sweden; INSERM, FRANCE

## Abstract

Metabolic complications are prevalent in individuals treated with cranial radiotherapy (CRT) for childhood acute lymphoblastic leukemia (ALL). The hypothalamus is a master regulator of endocrine and metabolic control. The aim of this study was to investigate whether the hypothalamic volume would be associated to metabolic parameters in ALL survivors. Thirty-eight (21 women) survivors participated in this study 34 years after diagnosis and with a median age of 38 (27–46) years. All were treated with a median CRT dose of 24 Gy and 11 years (3–13) of complete hormone supplementation. Comparisons were made to 31 matched controls. We performed analyses of fat mass, fat free mass, plasma (p)-glucose, p-insulin, Homa-Index (a measure of insulin resistance), serum (s)-leptin, s-ghrelin and of the hypothalamic volume in scans obtained by magnetic resonance imaging (MRI) at 3 Tesla. Serum leptin/kg fat mass (r = -0.4, P = 0.04) and fat mass (r = -0.4, P = 0.01) were negatively correlated with the HT volume among ALL survivors, but not among controls. We also detected significantly higher BMI, waist, fat mass, p-insulin, Homa-Index, leptin/kg fat mass and s-ghrelin and significantly lower fat free mass specifically among female ALL survivors (all P<0.01). Interestingly, s-ghrelin levels increased with time since diagnosis and with low age at diagnosis for childhood ALL. Our results showed that leptin/kg fat mass and fat mass were associated with a reduced HT volume 34 years after ALL diagnosis and that women treated with CRT after ALL are at high risk of metabolic abnormalities. Taken together our data suggest that the hypothalamus is involved in the metabolic consequences after CRT in ALL survivors.

## Introduction

Survival rate after childhood cancer has improved markedly and today more than 80% of patients with a pediatric malignancy will become 5-year survivors [1]. Nevertheless, survivors exposed to cranial radiotherapy (CRT) are at particularly high metabolic risk and survivors of childhood brain tumors with hypothalamic damage are at increased risk for obesity [2]. It is established that the largest childhood cancer group, the acute lymphoblastic leukemia (ALL) survivors, treated with CRT have Growth Hormone-deficiency (GH-deficency), obesity and lipid abnormalities [3]. Further, the metabolic hormones insulin and leptin, with receptors in the hypothalamus (HT) have shown resistance [3, 4] among the GH deficient ALL survivors [5, 6] and particularly among ALL women [3], suggesting a radiation-induced hypothalamic dysfunction. However, the effect of CRT on the HT in ALL survivors has not yet been studied.

The HT is a small complex area of the brain involved in endocrine function and energy homeostasis and consists of distinct nuclei including the infundibular nucleus (INF), the paraventricular nucleus (PVN), the dorsomedial nucleus (DMN) and the ventromedial nucleus (VMN) [7]. Destruction of these nuclei induces hyperphagia, hyperinsulinemia and weight gain [7] and these structures express high levels of leptin and ghrelin receptors [8, 9]. To our knowledge, there is only one study that investigated the hypothalamic volume in relation to metabolic risk; and healthy adolescent obese women had a smaller hypothalamic volume than their male counterpart [10]. Importantly, no difference in HT volume was shown between obese and lean subjects [10]. To the best of our knowledge no study has investigated the relationship between HT volume and metabolic risk in childhood cancer survivors.

Ghrelin levels are decreased in obese humans [11] and in craniopharyngeoma patients ghrelin levels decrease in parallel with the HT involvement by the tumor [12]. In contrast, ghrelin levels are increased in other populations with a possible hypothalamic dysfunction e. g. Prader Willi Syndrome [13], but have not been reported in ALL survivors.

We hypothesized that the HT might be affected after CRT in ALL patients and that its volume may have an impact on the persistent metabolic problems. Furthermore, based on the previously described sex differences, where ALL women were more affected than ALL men, regarding metabolic complications [3], we anticipated that metabolism-regulating peptides like insulin, leptin and ghrelin might be more affected in ALL women compared to ALL men.

## Material and Methods

### ALL survivors

From the South Region of Sweden there were 50 eligible ALL survivors and all of them had had pituitary hormone evaluation. All patients had received chemotherapy according to the common protocols of the Nordic countries [14] at the Children’s Hospital Lund in Sweden. All survivors were ≥18 years of age at the time of the study. Twelve survivors declined participation due to lack of time. Thus, we included 38 (76%) ALL survivors in the present study of metabolic risk factors. All 38 patients performed the magnetic resonance imaging (MRI) examination but due to claustrophobia or technical problems four were excluded, thus leaving 34 ALL that were included in the MRI evaluation. Median age at investigation was 38 (range min-max, 27–46) years and median time from CRT was 34 (23–42) years. All ALL survivors were found GH deficient after proper testing with the combination of insulin-tolerance test and GHRH-Arginine-test during adulthood [15]. They were all supplemented with GH 0.4 (0.2–0.8) mg/day during 11 (3–13) years, and 16% of the survivors were on thyroxin treatment and one survivor was treated with hydrocortisone for adrenal insufficiency. Ten ALL women had spontaneous and regular menstrual cycles, 3 women had primary amenorrhea and 8 women were using estroprogestative contraceptives. Six of the 14 ALL men had received radiation to the testes and were substituted with testosterone as intra muscular injections or gel (Table 1). One survivor among the ALL men had diabetes type 2. The ALL patients had 11 (3–13) years of complete hormone replacements.

**Table 1:** Characteristics including previous ALL treatment and hormone supplementation in 38 ALL survivors divided by gender with no significant difference in treatment modalities between gender.

	Men (n = 17)	Women (n = 21)
	Median (range)	Median (range)
**Current age (yr)**	38 (29–44)	39 (27–46)
**Age at CRT (yr)**	4 (1–17)	4 (1–13)
**Time from CRT (yr)**	33 (23–40)	35 (26–42)
**Target dose CRT (Gy)**	24 (18–30)	24 (20–25)
**Anthracycline (mg/m**^**2**^**)**	120 (80–540)	120 (40–480)
**Methotrexate dose it (mg/m**^**2**^**)**	62 (15–409)	60 (40–204)
**Methotrexate dose iv (mg/m**^**2**^**)**	1880 (30–4000)	3000 (3000–4000)
**Methotrexate iv (n)**	4	4
**Prednisolone dose (mg/m**^**2**^**)**	2147 (1633–7980)	2508 (1600–6682)
**Decadrone dose (mg/m**^**2**^**)**	370 (296–400)	335 (200–390)
**Spinal radiation (n)**	3	2
**GH during childhood (n)**	1	2
**GH therapy (dose)**	0.4 (0.2–0.5)	0.4 (0.2–0.5)
**GH therapy (years)**	10 (3.5–13)	11.5 (3–13)
**Testosterone substitution (n)**	6	0
**Thyroxine (n)**	2	4
**Cortisone substitution (n)**	1	0

Abbrevations: ALL, acute lymphoblastic leukaemia; CRT, cranial radiotherapy; n, numbers; yr; years, GH, growth hormone

### Control subjects

The 38 survivors (21 women) were matched with 31 control subjects (18 women) similar in age-, sex-, smoking habits and residence who were randomly selected from a computerized population register as previously shown [3].

### Study design

The present investigations were performed during a single day for each individual participant. The study was approved by the Lund University ethical committee in Sweden (DNR 2012/596). All participants gave written informed consent.

### Anthropological measurements

Assessments of body mass index BMI (kg/m^2^) was calculated as body weight (kilograms) divided by height (meters) squared. Waist (cm) was measured at the midpoint between the lower rib margin and the iliac crest. Body composition was assessed with dual-energy x-ray absorptiometry (DXA). Quality-assurance tests recommended by the manufacturer were performed using a total-body phantom. The CV from the scans was 0.4% for soft tissue. Data are expressed as estimated fat mass (kg) and fat free mass (kg).

### Assays

Blood samples were drawn in the morning, after an over-night fasting. Venous plasma (p)-glucose (mmol/L) was analyzed with a blood glucose analyzer (Hemocue AB, Ängelholm). P-Insulin (mIE/L) was measured with a competitive radioimmunoassay with intra and interassay CVs of 7,1% or less. Serum (s)—IGF-1 was measured with a chemiluminescent immunoassay. The normal range was 71–239 μg/L in subjects aged 31–42 years (interassay coefficient of variation (CV)%, 8% at the level of 30 μg/L and 8% at the level of 239 μg/L). The homeostasis model of assessment (Homa) index was calculated as the product of the fasting p-insulin level (microU/mL) and the fasting p-glucose level (mmol/L), divided by 22.5. Leptin was measured with a commercially available RIA-kit from Millipore Corporation, Linco Research, Inc. The within, between and total CV% were determined to be approximately 4.5%, 2.3% and 5.1% respectively at 3.9 μg/L. The corresponding figures at 20.5 μg/L were 5.1%, 2.1% and 5.5%. Ghrelin (total) was measured with a commercially available RIA-kit from Millipore Corporation, Linco Research, Inc. The analytical sensitivity of the method was estimated to be typically better than 100 ng/L. The within, between and total CV% were determined to be approximately 6.4%, 2.5% and 6.9% respectively at 550 ng/L. The corresponding figures at 1229 ng/L were 3.9%, 5.7% and 6.9%.

### Neuroimaging protocol

Imaging sequences were acquired on a 3-Tesla MR scanner (MAGNETOM Skyra, Siemens healthcare, Erlangen, Germany) using a 20 channel head/neck receive coil. Participants were in the supine position and their head was padded to minimize patient movement. For volumetric measurements axial T1-weighted MPRAGE images were acquired (acquisition parameters: 1 mm isotropic resolution, TE 3 ms, TR 1900 ms, flip angle 9).

### Measurement of the hypothalamic volume

**Fig 1:**
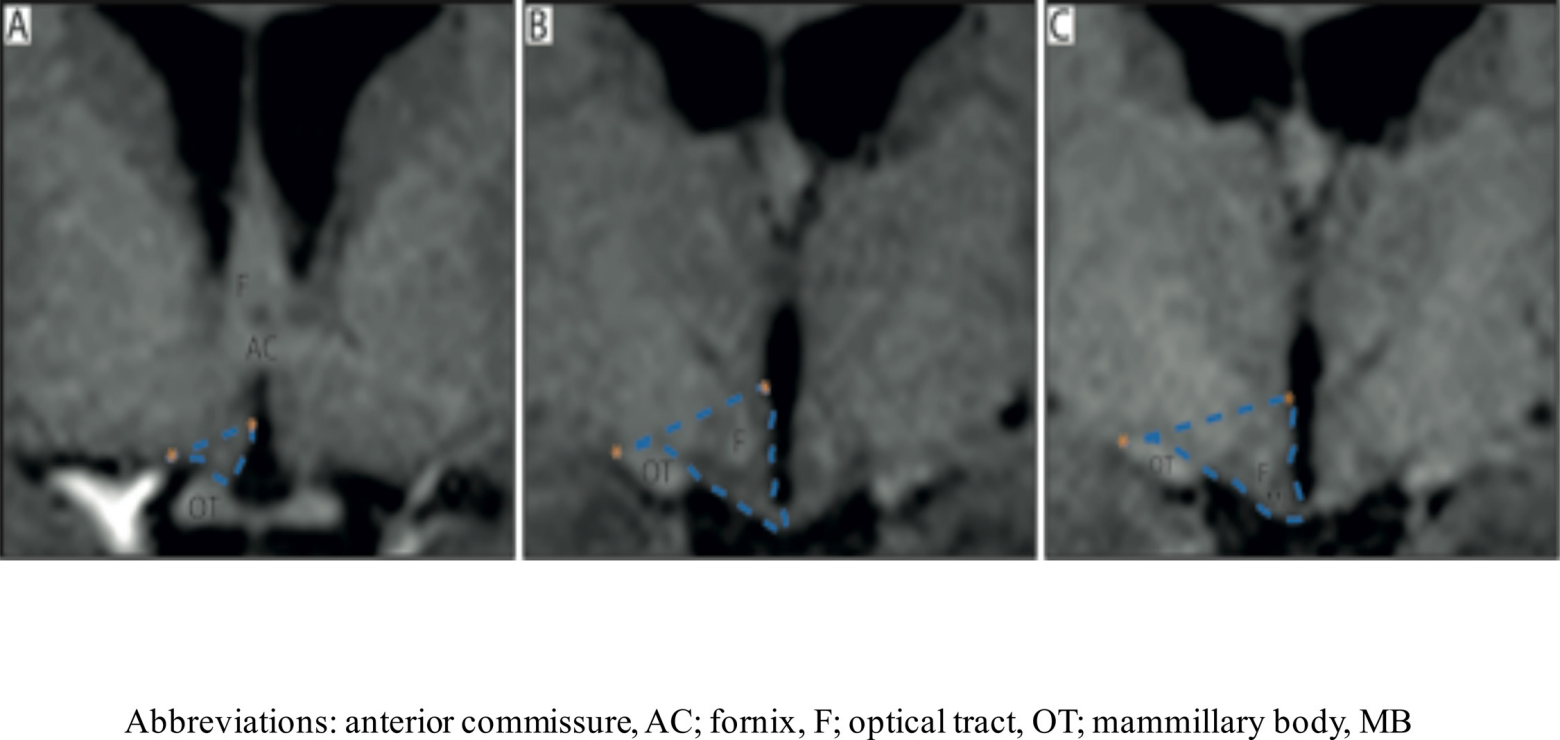
Overview of the delineation of the HT. Overview of the boundaries used to delineate the HT in T1-weighted MRI acquired at 3T according to the procedure established by Gabery et al., 2014 [17]. **A-C** represent the hypothalamic region in a coronal plane from rostral to caudal direction. The blue dashed lines illustrate how the hypothalamic region was delineated. Landmarks such as the hypothalamic sulcus and the lateral or medial edge of the optical tract represented by orange stars were identified for the delineation. A straight line between these two points was drawn to set the superior/lateral border of the area. The optical tract was excluded in all slides.

The volumetric measurements of the HT were performed manually with the ANALYZE 10.0 software package (Biomedical Imaging Resource, Mayo foundation, Rochester, MN) using a pen and digitized drawing pad. It was carried out on T1-weighted images, which were pre-processed by acquiring cubic spline interpolation and a resizing of original voxel size to 0.5 x 0.5 x 0.5 mm. The delineation of the HT was made using the anatomical borders recently established by Gabery et al. [16]. Briefly, the delineation was performed on a rostral to caudal axis on coronal images. The first anterior section of the HT was set once the optic chiasm was visualized being attached to the ventral part of the septal area at bregma level 1.3 mm (according to the Atlas of Human brain by Mai et al., 2008) [17]. A superior border was drawn as a straight line from the hypothalamic sulcus to the most lateral point of the optic tract throughout the region. The inferior border was set at the junction to the optical chiasm for anterior sections, and once the chiasm no longer was visible in more posterior sections, the border was set at the level of the infundibular nucleus, and defined by the border to cerebrospinal fluid. The final posterior border was set at the level when the fornix appeared to be merged with the mammillary nucleus at bregma level 9.3 mm. The optical tract was excluded in all slices. The intra-rater reliability of the method established in Gabery et al 2015 was high [18]. The coefficient of variation (CV: standard deviation of the hypothalamic volume /mean of the hypothalamic volume x 100) was estimated for a number of cases to assess the accuracy and reproducibly of the hypothalamic segmentation. A CV value < 4% was considered acceptable. The inter-rater reliability for this method was also high, with an intra-class correlation coefficient (ICC) of 0.937 [16]. A single person blinded to the identity of the cases carried out all hypothalamic segmentation (Fig 1).

### Measurement of the total intracranial volume (ICV)

The total intracranial volume (ICV) was calculated for each subject to perform adjustments of the hypothalamic volume between subjects with different head sizes. The INSECT (Intensity-Normalized Stereotaxic Environment for Classification of Tissues) [18, 19] algorithm was applied to estimate tissue fraction volumes (grey matter (GM), white matter (WM), and cerebrospinal fluid (CSF)). Initially, individual MRI volumes are corrected for intensity non-uniformity using the N3 algorithm [20]. This is a fully automated technique which maximizes the entropy of the intensity histogram and can be applied to any pulse sequence, field strength or MR scanner. Subsequently, the images are segmented into GM, WM and CSF using an artificial neural network classifier termed INSECT. In addition, global GM, WM and CSF volumes are computed. The corresponding fractions (relative volumes) are calculated by dividing the volume of a tissue class by the total intracranial volume (ICV = GM+WM+CSF).

### Statistics

Data are presented as median and range (min-max). Differences between ALL patients and matched population controls were compared using Mann-Whitney U test. Bivariate correlations were assessed using Spearman rank correlation coefficient. A P-value < 0.05 was regarded as statistically significant. We used SPSS version 21.0 for the statistical analysis.

## Results

### Hormone levels in 38 ALL survivors and matched control subjects

We found no significant differences between ALL survivors and controls in serum Insulin Growth Factor 1 (s-IGF-1) 216 (81–265) *vs* 189 (78–284) μg/L, Thyroid Stimulating Hormone (s-TSH) 1.9 (0.01–3.6) *vs* 1.9 (0.6–4.9) mIE/L, free T4 15 (12–25) *vs* 16 (12–22) pmol/L, p-cortisol 449 (182–1040) *vs* 445 (86–826) nmol/L, s-testosterone in men 19.5 (9–98) *vs* 9.8 (0.5–27.1) nmol/L, s-estradiol in women 266 (81–1028) *vs* 108 (40–548) pmol/L (all P>0.05).

### Metabolic risk factors in 38 ALL survivors compared to matched control subjects

We detected significantly higher BMI (26.7 *vs* 24.0 kg/m^2^) (P = 0.05), p-glucose (5.2 vs 4.9) (P = 0.005) p- insulin (10 *vs* 6 mIE/L) (P = 0.008), Homa-Index (0.14 *vs* 0.07) (P = 0.002), s-leptin (20.5 *vs* 11.5) (P = 0.07), leptin/kg fat mass (0.78 *vs* 0.56) (P = 0.02) and s-ghrelin (1165 *vs* 962 ng/L) (P = 0.04) among ALL survivors compared to matched controls. We detected no significant difference in weight (70.2 *vs* 72 kg) (P>0.05), waist (89.5 *vs* 83,5 cm) (P>0.05), fat mass (27.8 *vs* 23.1) (P>0.05) or fat free mass (42.8 *vs* 44.0 kg) (P>0.05).

### Gender effects on metabolic risk factors after CRT treatment of ALL

We were first interested in investigating the effect on the metabolic risk factors in the female group that consisted of 21 women treated for ALL and 18 control women (Table 2).

**Table 2:** Antrophological measurements and peptides in 21 ALL women 34 years after treatment with CRT compared to 18 healthy matched women.

	ALL women (n = 21)	Control women (18)	P
	Median (range)	Median (range)	
**Weight (kg)**	69.5 (38.7–97.7)	65 (53–95)	0.01
**Height (m)**	1.58 (1.46–1.79)	1.70 (1.60–1.73)	0.001
**BMI (kg/m**^**2**^**)**	27.9 (18.1–39.1)	22.6 (20–33)	0.002
**Waist (cm)**	89 (73–110)	79 (69–105)	0.002
**Fat mass (kg)**	29.9 (12.97–55.26)	22.4 (15.65–48.50)	0.001
**Fat free mass (kg)**	35.4 (26–48.90)	41.6 (35.51–46.74)	0.002
**P-Glucose (mmol/L**	5.2 (4.1–6.5)	4.9 (4.1–6.0)	>0.05
**P-Insulin (mIE/L)**	10 (2–31)	6 (4–16)	0.003
**Homa-index**	0.15 (0.02–0.46)	0.07 (0.04–0.19)	0.04
**S-Leptin (**μ**g/L)**	33 (13–90)	13 (7.3–40.0)	<0.001
**S-Leptin/kg fatmass**	1.09 (0.74–1.91)	0.6 (0.45–0.96)	<0.001
**S-Ghrelin (ng/L)**	1560 (556–3670)	993 (585–1710)	0.01

Abbrevations; ALL, acute lymphoblastic leukaemia; CRT, cranial radiotherapy BMI, body mass index; p, plasma; s, serum

We then investigated the effect on the metabolic risk factors in the male group that consisted of 17 ALL men and 14 control men. However, the metabolic differences detected in the female group were not present in the male group (Table 3).

**Table 3:** Antrophological measurements and peptides in 17 ALL men 34 years after treatment with CRT compared to 14 healthy matched men.

	ALL men (n17)	Controls men (n = 14)	
	Median (range)	Median (range)	>0.05
**Weight (kg)**	73 (58–123)	88 (67–149)	>0.05
**Heigth (cm)**	1.73 (1.62–1.91)	1.78 (1.70–1.88)	0.03
**BMI (kg/m**^**2**^**)**	25.4 (16.7–36.1)	26.4 (21–49)	>0.05
**Waist (cm)**	89 (74.5–119)	95 (81.5–120)	>0.05
**Fat mass (kg)**	22.45 (3.25–53.73)	26.21 (10.2–49.57)	>0.05
**Fatfree mass (kg)**	53.41 (43.95–73)	57.43 (47.32–63.59)	>0.05
**P-Glucose (mmol/L)**	5.1 (4.1–13.6)	4.8 (3.7–5.5)	0.02
**P-Insulin (mIE/L)**	10.5 (1–32)	8 (3–62)	>0.05
**Homa-index**	0.13 (0.01–1.07)	0.09 (0.03–0.78)	>0.05
**S-Leptin (**μ**g/L)**	7.2 (1.7–25)	7 (1.8–63)	> 0.05
**S-Leptin/kg fatmass**	0.34 (0.24–0.69)	0.30 (0.18–1.27)	>0.05
**S-Ghrelin (ng/L)**	966 (562–2920)	886 (592–1350)	> 0.05

Abbrevations; ALL, acute lymphoblastic leukaemia; CRT, cranial radiotherapy BMI, body mass index; p, plasma; s, serum

### Effects on the hypothalamic volume after CRT in ALL

**Fig 2:**
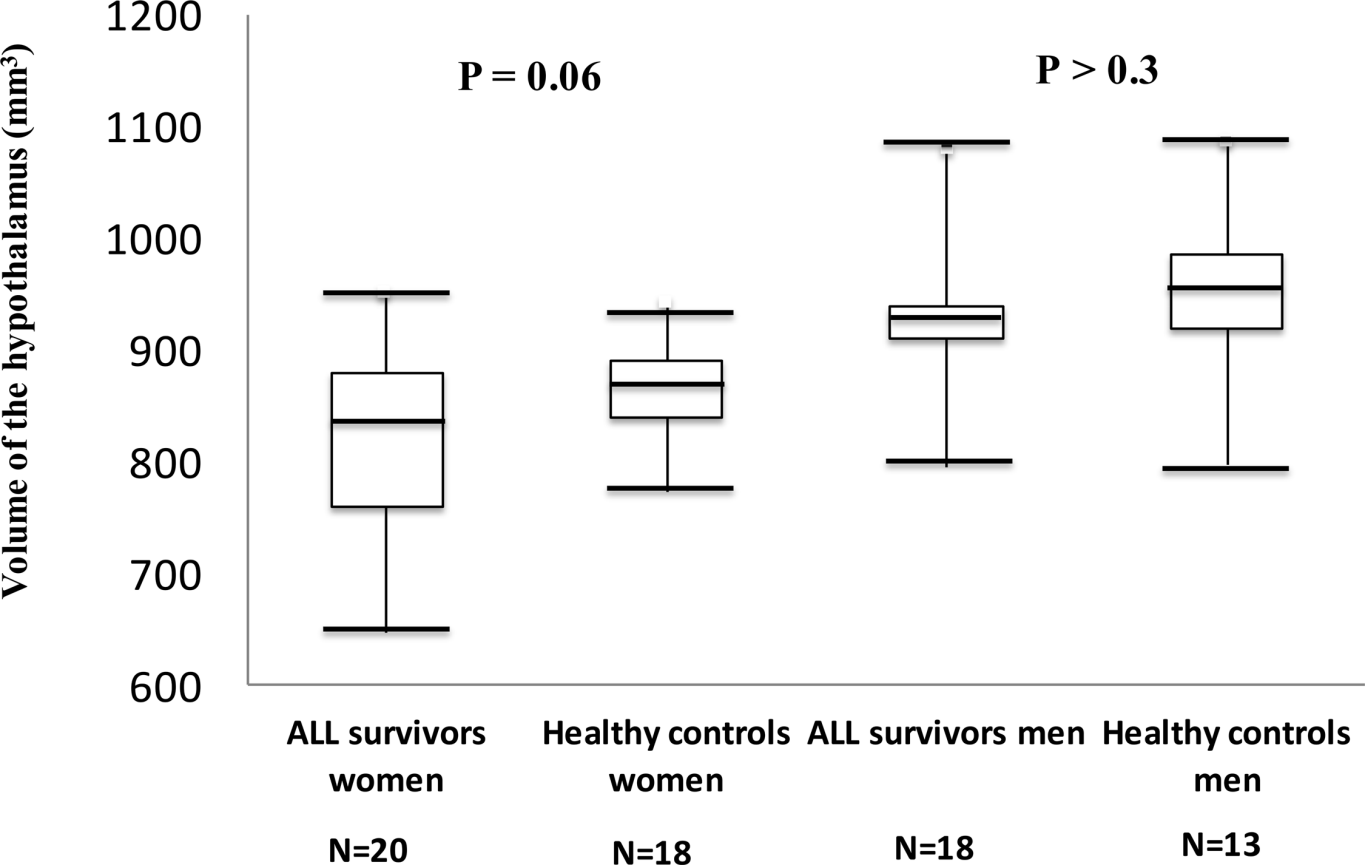
Hypothalamic volume in acute lymphoblastic leukemia (ALL) women and ALL men compared to gender matched controls. Boxplots illustrating the estimated hypothalamic volumes in ALL survivors compared to gender matched controls.

We estimated the HT volume in 20 women and 14 men treated for ALL compared to 32 controls. There was a trend of a smaller HT volume among ALL women compared to gender matched controls (846 (648–956) *vs* 869 (774–941) mm^3^, P = 0.06) (Fig 2). There was no significant difference in the HT volume between ALL men compared to control men (P>0.3) (Fig 2). There was a gender difference among ALL survivors with a significantly smaller HT volume in ALL women compared to ALL men (846 *vs* 936 mm^3^, P = 0.001), which was in accordance with a close to significant gender difference among the controls (869 *vs* 926 mm^3^, P = 0.06). We did not find a correlation between height and HT volume in ALL survivors (r = 0.2, P > 0.05) or in controls (r = 0.3, P > 0.05), and with no gender difference for this correlation (P > 0.05).

### Intracranial volume (ICV) and volume of HT corrected for ICV

ALL survivors had a reduced ICV compared to controls 1112 (868–1503) *vs* 1156 (465–1371) mm^2^, P = 0.005). There was no significant difference in volume of HT corrected for ICV between ALL survivors and controls (78 (64–98) *vs* 77 (59–1371), P>0.05). There was a significantly positive correlation of ICV to volume of HT (r = 0.7, P<0.001) in 34 ALL survivors, but not in controls (r = 0.2, P = 0.2).

### Associations between metabolic risk factors and the hypothalamic volume in 34 ALL survivors treated with CRT

**Fig 3:**
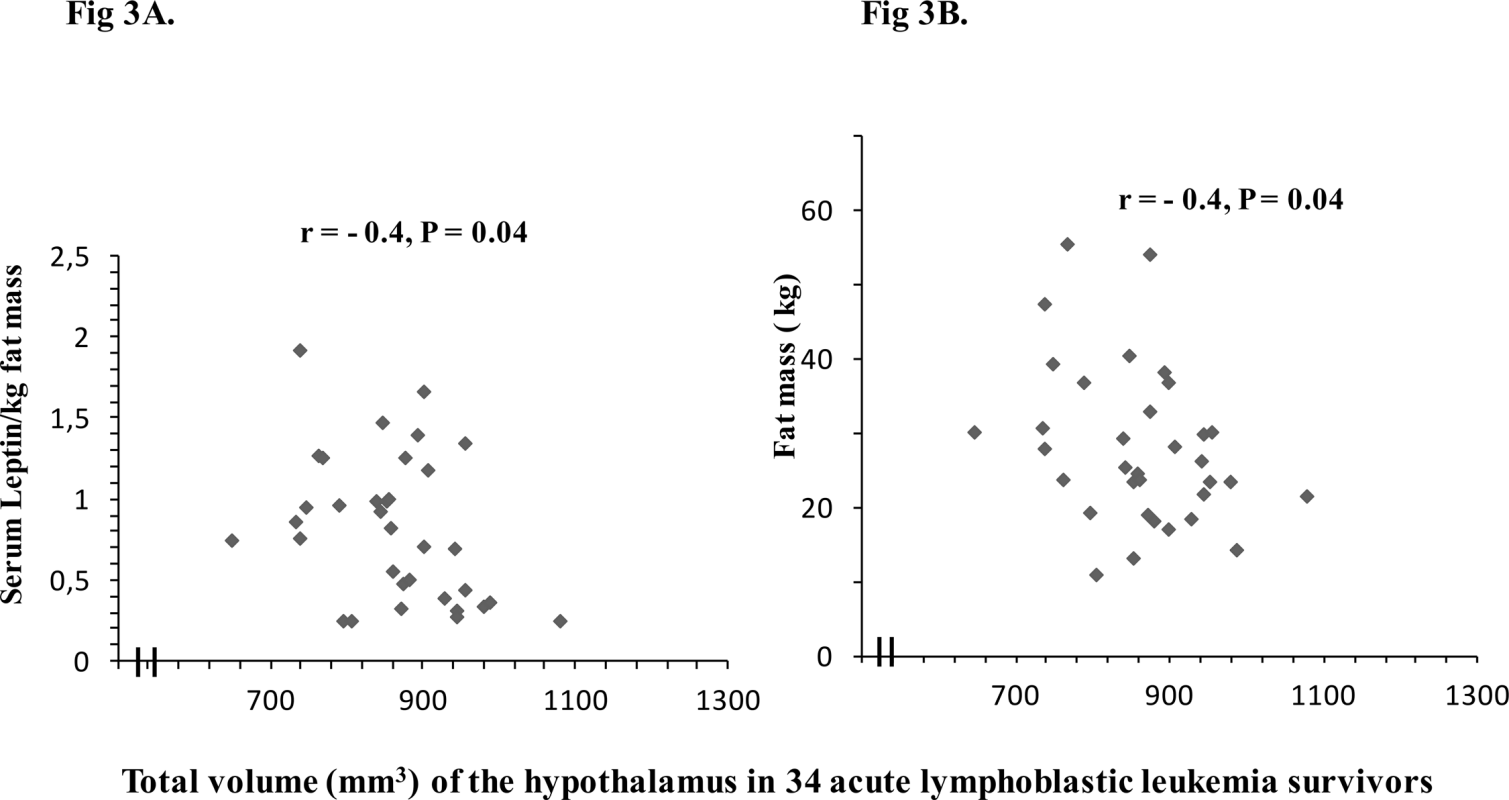
A. Relationship between leptin/kg fat mass and HT volume in 34 acute lymphoblastic leukemia (ALL) survivors. Spearman correlation coefficients (r) and levels of statistical significance (p) are shown. B. Relationship between HT volume and fat mass in 34 acute lymphoblastic leukemia (ALL) survivors. Spearman correlation coefficients (r) and levels of statistical significance (p) are shown.

There was a significantly negative correlation of leptin/kg fat mass to HT volume (r = -0.4, P = 0.04) in 34 ALL survivors (Fig 3A). There was a significantly negative correlation of fat mass to HT volume (r = —.4, P = 0.04) in 34 ALL survivors, but not in controls (Fig 3B). There were no correlations of age at ALL diagnosis or years since ALL diagnosis to HT volume (P>0.05).

### Associations between the metabolic risk factors in ALL survivors treated with CRT

**Fig 4:**
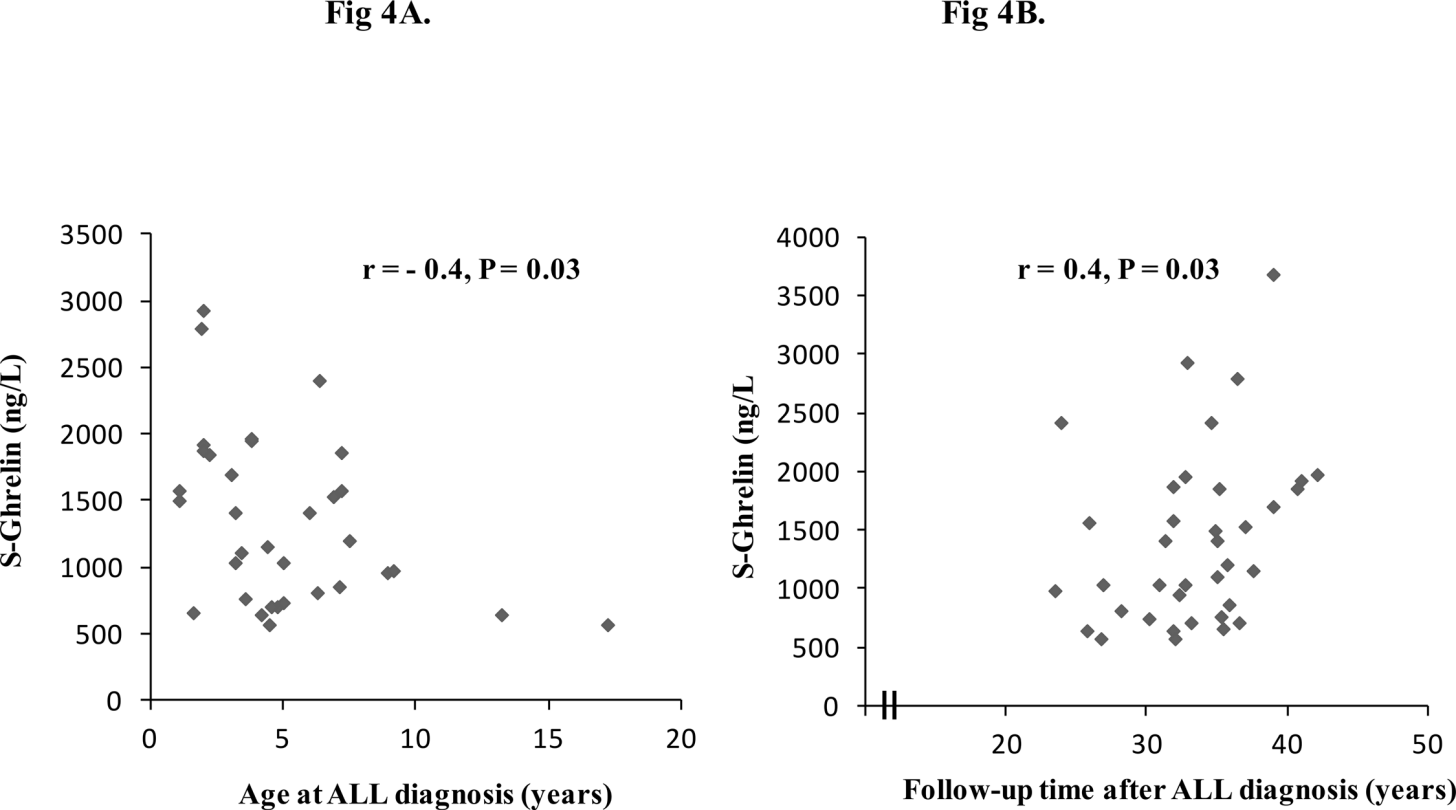
A. Relationship between serum ghrelin and age at acute lymphoblastic leukemia (ALL) diagnosis in 38 survivors. Spearman correlation coefficients (r) and levels of statistical significance (p) are shown. B. Relationship between serum ghrelin and follow-up time after acute lymphoblastic leukemia (ALL) diagnosis in 38 ALL survivors. Spearman correlation coefficients (r) and levels of statistical significance (p) are shown.

There was a significantly negative correlation of serum ghrelin to BMI in 38 ALL survivors (r = -0.5, P = 0.004, and in ALL women (r = -0.5, P = 0.02). No such correlation found shown in ALL men or in controls. There was a significantly negative correlation of p-insulin to s-ghrelin levels (r = -0.4, P = 0.01) in 38 ALL survivors. There was a significantly negative correlation of s-ghrelin to age at diagnosis (r = -0.4, P = 0 .03) (Fig 4A) and a positive correlation of s-ghrelin to follow-up time after diagnosis (r = 0.4, P = 0.03) (Fig 4B). HT volume in ALL survivors did not correlate significantly to levels of ghrelin (r = -0.2, P>0.05). There was no correlation between levels of s-IGF-1 or weight and s-ghrelin (P > 0.05).

## Discussion

This is the first report on measurements of the hypothalamic volume in childhood cancer survivors. The current study showed that 34 years after CRT and chemotherapy there was a significant association between increased levels of leptin/kg fat mass or fat mass to a reduced volume of the HT in ALL survivors. Furthermore, there was a trend of a smaller HT volume among only ALL women compared to control women. Although complete pituitary hormone substitution was provided at an average of 11 years, there were still metabolic complications with insulin resistance and increased fat mass in ALL women. Our data suggest a gender-specific increased sensitivity to hypothalamic dysfunction after CRT or chemotherapy. A possible mechanism contributing to the smaller HT volume could be CRT-induced toxicity to HT neurons important for the energy homeostasis and possibly the female brain might be more vulnerable to CRT. This is underscored by the fact that no gender difference was present in the dose of CRT, age at, or time since CRT, between ALL women and men in the present study (Table 1). We cannot however, exclude that the impaired metabolic risk among the ALL survivors have an impact on the volume of the HT. Particularly among the ALL women with the most affected BMI (median 27.9 (18.1–39.1) in comparison to controls. The high BMI among ALL women could have an impact on HT volume. However, also ALL men had a wide range of BMI (16.7–36.1, median 25.4), but without a reduction in HT volume.

Orexigenic neuropeptide populations express receptors for peripheral hormone signals such as insulin, leptin and ghrelin [7]. Agouti-related peptide (AgRP) and NPY neurons are situated in the INF and are part of the melanocortin system, which is critical for sensing a number of peripheral signals controlling food intake and energy homeostasis. Our finding of increased levels of leptin/kg fat mass or fat mass associated with a reduced HT volume among the ALL survivors indicate an hypothalamic involvement after CRT treatment, which was further supported by the lack of such correlation among the healthy controls. In animal experiments it was shown that hyperleptinemia contributes to central ghrelin resistance and a dysfunction in NPY/AgRP neurons [21]. This is in accordance with our present findings of hyper-leptinemia, which might be associated with the new finding of increased ghrelin levels in particularly female ALL patients. Furthermore, electrophysiological studies have shown that leptin inhibits the NPY neurons to respond to ghrelin [22]. These findings show the importance of the medial HT as a target for the actions of ghrelin on appetite and energy expenditure and the close relationship between leptin and ghrelin.

The importance of increased of serum ghrelin levels with the follow-up time and that children exposed to CRT at young age have increased serum ghrelin levels indicate that this condition aggravates with time after diagnosis and with young age at treatment. We showed previously an increase in BMI with follow-up time since diagnosis [23], but this cannot explain our result as there was a negative association between ghrelin levels and BMI, which means higher BMI in survivors with lower serum ghrelin.

Insulin is believed to play a role in regulating body weight through a direct central effect and has also been shown to increase leptin [24]. This relationship is the opposite of that seen with ghrelin. Insulin and ghrelin levels have been shown to correlate negatively in obese humans [12] and insulin seems to suppress ghrelin [25, 26]. This is in accordance with the present study showing a negative correlation between insulin and ghrelin levels. However, it is controversial whether ghrelin stimulates or inhibits insulin secretion [27, 28].

We found a significantly reduced volume of HT in ALL women compared to ALL men and also a close to significant difference between the female controls compared to male controls. This gender difference has been reported among obese adolescent females that had a smaller HT volume compared to their male counterparts [10]. However, studies have not shown a significant difference in HT volume between obese and lean subjects [10]. Thus, obesity *per se* may not affect the HT volume. We did not match the control group for BMI or height as these variables are considered outcome measures of the disease and treatment during childhood. Interestingly, women with type 2 diabetes mellitus compared to men with the same condition, have a greater vulnerability of the brain to metabolic-related damage [29] and a greater risk for cardiovascular disease [30]. It is well known that an increased metabolic risk is shown in patients with GH deficiency or hypothyroidism [31]. Thus, it has to be pointed out that in contrast to many other ALL studies [2, 4, 5] reporting an increased metabolic risk among the ALL patients, this is the only study where the ALL patients have been supplemented with all necessary hormones. Interestingly, after 11 years of complete hormone supplementation we show that female ALL patients and not the males are at increased metabolic risk.

ALL survivors in the present study were all GH deficient and on GH therapy [23] with normal s-IGF-1, but a GH to ghrelin feedback is unlikely to explain our findings. On the contrary, it has been reported that GH suppresses ghrelin levels in GH-deficient patients, supporting the hypothesis that a GH feedback inhibits ghrelin secretion [32, 33]. In addition, we did not find an association between serum levels of ghrelin and IGF-1.

The paraventricular nucleus (PVN) is dense in sex-steroid receptors [34], and sex steroids have a direct effect on obesity and adipose tissue [35]. Thus, one potential explanation for the differences in HT volume between men and women could in part be related to differences in LH and FSH or sex steroids, although this is unlikely as we did not find a difference in estrogen levels between the ALL women and their controls. A previous study [36] showed increased levels of LH during puberty in humans together with an increase in pituitary volume and also a correlation between LH levels and pituitary volume. However, they did not find an increased hypothalamic volume during puberty when GnRH and LH and FSH levels are known to increase.

ALL survivors in the present study have decreased adult height compared to healthy controls (Table 2 and Table 3) and height is known to be associated with brain volume in healthy subjects, suggesting that correcting brain volumes for head size is necessary when performing comparisons between subjects [37]. However, we did not find a correlation between height and HT volume in ALL survivors or in controls. We did find a reduced ICV in survivors compared to healthy controls and a positive correlation of HT volume to ICV, but *only* in survivors. In the present study, we also report ICV-corrected HT volume and we found no significant difference in HT volume between survivors and controls when taking ICV into account. We suggest that the reduced ICV in survivors may reflect a deranged neurodevelopment due to previous ALL treatment resulting in a smaller HT volume which also may affect its function.

This study focused on total volumetric measurements of the entire structure of the HT and the current techniques did not allow us to study specific nucleus of interest. Further, our small number of subjects is not sufficient to draw any definitive conclusions, but the ALL survivors in the present study are representative of the entire population of ALL patients treated with CRT and chemotherapy worldwide. Furthermore, we compared the ALL patients with age, gender matched controls from the general population.

In conclusion, 34 years after CRT and chemotherapy, increased levels of leptin/fat mass or fat mass was associated with a reduction of HT volume, suggesting that hypothalamic dysfunction may underlie these metabolic complications after ALL. The increase in serum ghrelin levels with time since diagnosis might mirror a progressive impairment after cranial radiotherapy in childhood. In spite of complete hormone supplementation, ALL women suffered from an increased metabolic risk. Future studies are needed to confirm and clarify the significance of our findings and a more detailed imaging of the HT might identify the specific hypothalamic nuclei affected. No treatment is currently available for these hypothalamic problems, but the mainstay is tailored healthy life-style support and proper hormone replacement.
